# Surface electromyography to quantify neuro-respiratory drive and neuro-mechanical coupling in mechanically ventilated children

**DOI:** 10.1186/s12931-023-02374-w

**Published:** 2023-03-13

**Authors:** Alette A. Koopman, Jefta van Dijk, Eline Oppersma, Robert G. T. Blokpoel, Martin C. J. Kneyber

**Affiliations:** 1 Division of Paediatric Critical Care Medicine, Department of Paediatrics, Beatrix Children’s Hospital, University Medical Center Groningen, University of Groningen, Hanzeplein 1, 9713 GZ Groningen, The Netherlands; 2 Cardiovascular and Respiratory Physiology Group, TechMed Centre, University of Twente, Enschede, The Netherlands; 3 Critical Care, Anaesthesiology, Peri-Operative & Emergency Medicine (CAPE), University of Groningen, Groningen, The Netherlands

**Keywords:** Children, Mechanical ventilation, Neuro-respiratory drive, Neuro-mechanical coupling, Surface electromyography, Pressure support

## Abstract

**Background:**

The patient’s neuro-respiratory drive, measured as electrical activity of the diaphragm (EAdi), quantifies the mechanical load on the respiratory muscles. It correlates with respiratory effort but requires a dedicated esophageal catheter. Transcutaneous (surface) monitoring of respiratory muscle electromyographic (sEMG) signals may be considered a suitable alternative to EAdi because of its non-invasive character, with the additional benefit that it allows for simultaneously monitoring of other respiratory muscles. We therefore sought to study the neuro-respiratory drive and timing of inspiratory muscles using sEMG in a cohort of children enrolled in a pediatric ventilation liberation trial. The neuro-mechanical coupling, relating the pressure generated by the inspiratory muscles to the sEMG signals of these muscles, was also calculated.

**Methods:**

This is a secondary analysis of data from a randomized cross-over trial in ventilated patients aged < 5 years. sEMG recordings of the diaphragm and parasternal intercostal muscles (ICM), esophageal pressure tracings and ventilator scalars were simultaneously recorded during continuous spontaneous ventilation and pressure controlled-intermittent mandatory ventilation, and at three levels of pressure support. Neuro-respiratory drive, timing of diaphragm and ICM relative to the mechanical ventilator’s inspiration and neuro-mechanical coupling were quantified.

**Results:**

Twenty-nine patients were included (median age: 5.9 months). In response to decreasing pressure support, both amplitude of sEMG (diaphragm: *p* = 0.001 and ICM: *p* = 0.002) and neuro-mechanical efficiency indices increased (diaphragm: *p* = 0.05 and ICM: *p* < 0.001). Poor correlations between neuro-respiratory drive and respiratory effort were found, with R^2^: 0.088 [0.021–0.152].

**Conclusions:**

sEMG allows for the quantification of the electrical activity of the diaphragm and ICM in mechanically ventilated children. Both neuro-respiratory drive and neuro-mechanical efficiency increased in response to lower inspiratory assistance. There was poor correlation between neuro-respiratory drive and respiratory effort.

***Trial registration*:**

ClinicalTrials.gov ID NCT05254691. Registered 24 February 2022, registered retrospectively.

**Supplementary Information:**

The online version contains supplementary material available at 10.1186/s12931-023-02374-w.

## Background

Acute respiratory failure necessitating mechanical ventilation (MV) is one of the main indications for pediatric intensive care unit (PICU) admissions. Although the technique of MV itself is lifesaving for patients with impaired gas exchange, it can also cause lung and diaphragm injury by a variety of interacting mechanisms [1, 2]. Whereas lung injury mainly comes from mechanical stress and strain caused by the ventilator (ventilator-induced lung injury), there is also mounting evidence that spontaneous breathing during especially moderate-to-severe disease plays a role in the progression of lung injury, a paradigm known as patient self-inflected lung injury (P-SILI). Vigorous spontaneous breathing causes increased transpulmonary pressure swings, thereby amplifying lung damage [3–7]. It is commonly accepted to allow for spontaneous breathing in mechanically ventilated patients to protect the respiratory muscles. However, this must be balanced against the risk for P-SILI. Ventilator-induced diaphragmatic dysfunction refers to diaphragm atrophy and injury, occurring mainly through overassistance myotrauma (i.e., excessive unloading of the respiratory muscles), leading to a reduction or even suppression of the neuro-respiratory drive [8, 9]. Oppositely, insufficient ventilatory support failing to adequately unload respiratory muscles (i.e., excessive respiratory muscle loading), may cause inflammation and injury leading to underassistance myotrauma [8]. In critically ill adults, both types of myotrauma have been linked to prolonged duration of MV, prolonged ICU admission and a higher risk of complications including reintubation and need for tracheostomy [10].

Individualized monitoring of respiratory effort is necessary for the delivery of lung and diaphragm protective ventilation. Esophageal pressure (Pes) manometry is considered the gold standard for measuring patient inspiratory effort and transpulmonary driving pressure [11, 12]. However, accurate Pes monitoring is invasive, requires correct catheter positioning and an individualized balloon filling volume making it a cumbersome monitoring tool [11]. Monitoring the electrical activity of the diaphragm (EAdi) using a specifically designed esophageal catheter, represents the patient’s neuro-respiratory drive and correlates strongly with the transpulmonary driving pressure [13–15]. However, monitoring EAdi is limited to only one ventilator brand in clinical practice. The invasive nature of esophageal pressure manometry and EAdi monitoring may be perceived as challenging especially in small children. We propose that transcutaneous (surface) monitoring of respiratory muscle electromyographic (sEMG) signals may be considered a good alternative to EAdi because of it is non-invasive design, plus that it also allows for simultaneously monitoring of other respiratory muscles. We therefore sought to study the neuro-respiratory drive, timing of inspiratory muscles and neuro-mechanical coupling using sEMG in a cohort of children enrolled in a pediatric ventilation liberation trial.

## Materials and methods

### Study population

This study is a secondary analysis of data from mechanically ventilated children enrolled in a randomized cross-over trial investigating pediatric ventilation liberation. The study was performed in the 20-bed tertiary medical-surgical PICU of the Beatrix Children’s Hospital, University Medical Center Groningen (Groningen, the Netherlands). Subjects < 5 years of age deemed ready for weaning from invasive MV by their treating clinician were eligible for inclusion. Those with congenital or acquired neuromuscular disorders or paralysis of the diaphragm, severe traumatic brain injury (i.e., Glasgow Coma Score < 8), uncorrected congenital heart disorder, chronic lung disease and severe pulmonary hypertension were excluded. The Institutional Review Board approved the study (NL38361.042.11), and written informed consent was obtained from the parents or legal caretakers.

### Study protocol

In the parent study, the effects of two different ventilator modes and down tapering the level of pressure support (PS) were studied [16]. Briefly, subjects were randomly assigned to first a 10 min period in a continuous spontaneous ventilation (CSV) mode (i.e., PS ventilation) or pressure controlled-intermittent mandatory ventilation (PC-IMV) (i.e., pressure controlled assist/control [PC/AC]) with a low mandatory breath rate (25% of the set rate prior to study enrollment). Subsequently, subjects were crossed over to the other randomization arm. Afterwards, subjects were switched to PS ventilation. Then the PS level was decreased on three consecutive steps by 2 cm H_2_O. Positive end-expiratory pressure (PEEP) and fraction of inspired oxygen (FiO_2_) remained constant throughout the study.

### Data acquisition

sEMG recordings, Pes manometry and ventilator scalars were acquired at baseline and subsequently during each intervention according to the randomization outcome [16]. Data was recorded for 5 min during stable breathing.

Electrical activity of the diaphragm and parasternal intercostal muscles (ICM) was measured transcutaneous using pairs of single Ag/AgCl electrodes (EasyTrode TM Pre gelled Electrodes, Multi Bio Sensors Inc, El Paso, USA). Two electrodes were placed bilaterally at the costo-abdominal margin in the nipple line, and an electrode at both the left and right second intercostal space. A common electrode was placed at the sternal level [17]. sEMG was recorded using the Porti-16 data acquisition system (22 bits, TMSi; The Netherlands) with unipolar electrophysiological channels (71.5 nV/bit, gain: 20). An age-appropriate esophageal balloon catheter (Avea SmartCath 6 or 8 Fr, Vyaire, Mettawa, III, USA) was positioned in the lower 1/3 of the esophagus and connected to the Bicore II (Vyaire, Mettawa, III, USA). Correct position was verified by negative pressure deflections during spontaneous breathing and/or chest radiography that was done for other indications [18]. Esophageal balloon volume was titrated up to a maximum of 1.6 ml (pediatric catheter) or 2.6 ml (adult catheter). Optimal balloon volume was achieved by determining the volume with the Pes maximum amplitude.

A proximal flow sensor (VarFlex™, Vyaire, Mettawa, III, USA) was used to measure flow and Vt near the Y-piece of the endotracheal tube. Ventilator scalars were acquired using the ventilator’s analog output port. All signals were digitized with a sample frequency of 1024 Hz and stored offline (Polybench, Applied Biosignals GmbH, Weener, Germany).

Patient characteristics (gender, age, weight, 24-h Pediatric RISK of Mortality (PRISM) III score, admission diagnosis and endotracheal tube size) were obtained to characterize the study population [19].

### Offline signal processing and parameter calculation

The recorded sEMG signals of both diaphragm and ICM were processed as described previously [20].

We visually selected a period of 30 consecutive breaths free of artifacts from each series of measurements. Onset, peak, and termination of inspiratory muscle activity were determined in sEMG signals as reported by us previously [20]. The neuro-respiratory drive was quantified by the following breath-by-breath parameters, normalized to muscle activity at baseline measurement: maximal electrical activity (peak activity, EMG_peak_), the amplitude (the difference between the peak and tonic activity, EMG_amp_), the integral of EMG signal over time during the inspiration multiplied with respiratory rate (area under curve, EMG_AUC_/min), and mean electrical activity during a whole breath (mean activity, EMG_mean_). To evaluate the timing of inspiratory muscles relative to the MV, trigger and cycle times were determined as described previously [20]. The tidal esophageal peak-to-through during inspiration (Pes_amp_) was calculated. The esophageal pressure time product (PTP) was calculated by the integral of Pes over time during inspiration multiplied by respiratory rate. Expiratory tidal volume, respiratory rate and inspiratory time were calculated from the proximal flow signal. The neuro-mechanical coupling, i.e., the relationship between Pes and sEMG was analyzed by calculating indices for neuro-mechanical efficiency (NME) (i.e., Pes_amp_/EMG_amp_, and PTP/ EMG_AUC_/min).

### Statistical analysis

Statistical analyses were performed using Prism 5 (Graphpad software, San Diego, CA, USA) and Matlab R2018a (Mathworks, Natick, MA, USA). The Shapiro–Wilk test was used to test data for normality. Descriptive data were expressed as median [interquartile range (IQR)] or percentage (%) of total. The neuro-respiratory drive, neuro-mechanical coupling, and timing of the inspiratory muscles at each measurement series were compared using the Wilcoxon signed rank test. To assess if the changes in PS level induced significant changes in neuro-respiratory drive, neuro-mechanical coupling, and timing of the inspiratory muscles, one-way analysis of variance for repeated measures was performed (Skillings-Mack test). The relationship between neuro-respiratory drive and Pes-derived data (effort) in each ventilatory condition was assessed. For both inspiratory muscles, the correlation between EMG_amp_ and Pes_amp_, and between EMG_AUC_/min and PTP were determined using the determination coefficient *R*^2^. A *p* value < 0.05 was considered statistically significant.

## Results

Thirty-six subjects were enrolled in the parent study. sEMG recordings from seven subjects were excluded from analysis because of missing data (N = 1) and inability to detect inspiratory sEMG activity in both diaphragm and ICM due to crosstalk—i.e. electrical muscle activity of adjacent muscles (N = 4) or due to electrical interference (N = 2). Thus, 29 subjects (19 boys and 10 girls) with a median age of 5.9 [1.3–14.1] months and weight 6.8 [4.9–10.0] kg were included (Table 1). The majority of the patients was admitted for primary respiratory failure (86.2%).

**Table 1:** Patient characteristics

	Subjects
	(*n* = 29)
Age (months)	5.9 [1.3 to 14.1]
Sex male/female	19/10
Weight (kg)	6.8 [4.9 to 10.0]
PRISM III score	3.0 [1.0 to 4.25]
PIM II score	− 4.38 [− 4.66 to 3.92]
Admission diagnosis (*n*)	
Respiratory	25
Postoperative	2
Upper airway obstruction	2
Respiratory disease (*n*)	
Healthy lungs	4
Obstructive disease	1
Restrictive disease	4
Obstructive + restrictive	20
Duration of MV (days)	5.0 [3.3 to 6.9]
HFOV (*n*)	11
Duration of HFOV (d)	2.2 [1.4 to 2.6]
Length of PICU stay (d)	7.0 [5.1 to 9.5]
Extubation failure (*n*)	3

*PRISM III* 24-h Pediatric Risk of Mortality III, *PIM II* Pediatric Index of Mortality II, *MV* mechanical ventilation, *HFOV* high frequency oscillation ventilation

Data are shown in number or median [interquartile range]

### Neuro-respiratory drive

**Fig. 1:**
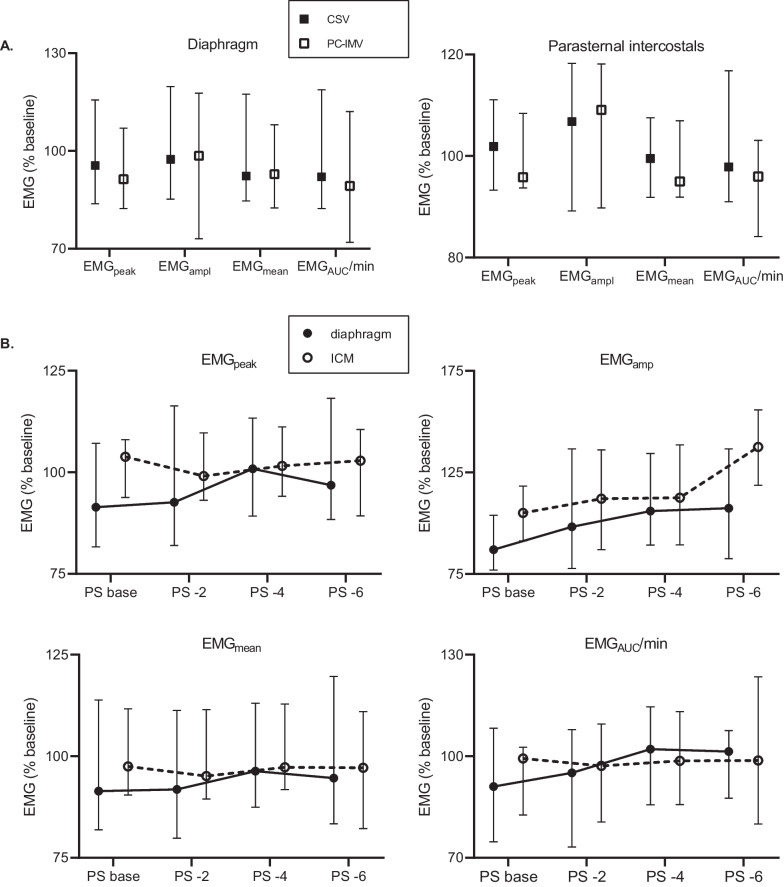
Inspiratory muscle activity of diaphragm and parasternal intercostal muscle (ICM) during **A** two different weaning methods and **B** reduction of pressure support (PS) level. The electromyography (EMG) values are normalized to activity during baseline measurement. Values are depicted as median (interquartile range). The number of subjects in which EMG parameters could be determined differed per muscle and ventilation mode or PS level. Median number of subjects, for the diaphragm *N* = 29 and ICM *N* = 24. *CSV* continuous spontaneous ventilation, *EMG*_*peak*_ peak EMG activity, *EMG*_*amp*_ EMG activity amplitude, *EMG*_*mean*_ mean EMG activity level during one breath, *EMG*_*AUC*_*/min* integral of EMG signal over time during the inspiration multiplied with the respiratory rate, *PC-IMV* pressure-controlled intermittent mandatory ventilation

Figure 1A displays the neuro-respiratory drive (i.e., EMG_peak_, EMG_amp_, EMG_mean_ and EMG_auc_/min) for the whole cohort and stratified by ventilator mode (i.e., PS ventilation or PC-IMV). For the whole cohort, the neuro-respiratory drive to the diaphragm was estimated by a median EMG_peak_ of 95.5% and 91.3%, median EMG_ampl_ of 97.4% and 98.5%, median EMG_mean_ of 92.3% and 92.9%, and median EMG_AUC_/min 92.0% and 89.3% during PS ventilation and PC-IMV, respectively. The neuro-respiratory drive to the ICM was described by a median EMG_peak_ of 101.9% and 95.8%, median EMG_ampl_ of 106.8% and 109.1%, median EMG_mean_ of 99.5% and 95.0%, and median EMG_AUC_/min 97.9% and 96.0% during PS ventilation and PC-IMV, respectively. The neuro-respiratory drive estimates were comparable between the two ventilation modes. EMG_amp_ changed with decreasing PS in both the diaphragm (median EMG_amp_ 87.0, 98.2, 105.9, and 107.4% of baseline for PS base, -2, - 4, and - 6 cm H_2_O, respectively; *p* = 0.001) and ICM (median EMG_amp_ 105.1, 112.1, 112.6, and 137.6% of baseline for PS base, -2, - 4, and - 6 cmH_2_O, respectively; *p* = 0.002) (Fig. 1B). Similar patterns were observed for diaphragmatic EMG_peak_ and EMG_auc_/min (median EMG_peak_ 91.4, 92.6, 100.9, and 96.8% of baseline for PS base, -2, -4, and -6 cm H_2_O, respectively; *p* = 0.02, and median EMG_auc_/min 91.0, 95.1, 102.1, and 101.4% of baseline for PS base, -2, -4, and -6 cmH_2_O, respectively; *p* = 0.03). The other neuro-respiratory drive estimates remained constant at various PS levels (*p* > 0.05). Absolute values of neuro-respiratory drive estimates are shown in Table S1 of Additional file 1.

### Timing of respiratory muscles

**Fig. 2:**
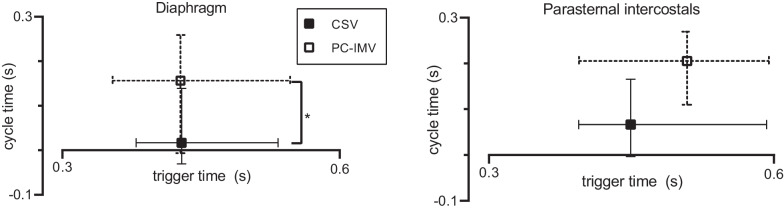
Trigger and cycle times of diaphragm and parasternal intercostals in two weaning approaches. Values are depicted as median (interquartile range). *Significant difference between two weaning approaches (*p* < 0.05). *CSV* continuous spontaneous ventilation, *PC-IMV* pressure-controlled intermittent mandatory ventilation

For the whole cohort, median diaphragmatic trigger time was 0.429 s during PS ventilation and 0.428 s during PC-IMV, and median ICM trigger time was 0.449 s during PS ventilation and 0.508 s during PC-IMV. Trigger times were not significantly different between PS ventilation and PC-IMV (*p* > 0.05). An increased cycle time during PC-IMV compared to PS ventilation was found for both diaphragm (median trigger time 0.017 and 0.157 s during PS ventilation and PC-IMV, respectively; *p* = 0.04) and ICM (median trigger time 0.066 and 0.205 s during PS ventilation and PC-IMV, respectively; *p* = 0.06). Trigger and cycle times of both inspiratory muscles are graphically depicted in Fig. 2. We did not observe any changes in trigger or cycle times when PS was reduced (Table 2). There were no timing differences between diaphragm and ICM within one ventilation condition.

**Table 2:** Trigger and cycle times of diaphragm and parasternal intercostal muscles at different pressure support levels

	PS base	PS-2	PS-4	PS-6
Diaphragm				
Trigger time (s)	0.42 [0.35 to 0.48]	0.43 [0.36 to 0.55]	0.38 [0.28 to 0.49]	0.41 [0.35 to 0.51]
Cycle time (s)	0.03 [− 0.08 to 0.08]	0.06 [− 0.01 to 0.12]	0.03 [− 0.02 to 0.10]	0.03 [− 0.04 to 0.14]
ICM				
Trigger time (s)	0.43 [0.38 to 0.56]	0.49 [0.39 to 0.52]	0.47 [0.37 to 0.55]	0.43 [0.38 to 0.50]
Cycle time (s)	0.08 [0.00 to 0.17]	0.06 [− 0.01 to 0.13]	0.05 [0.02 to 0.15]	0.04 [0.00 to 0.12]

*ICM* parasternal intercostal muscles, *PS* pressure support level

Data is shown as median [interquartile range]

### Neuro-mechanical coupling

**Fig. 3:**
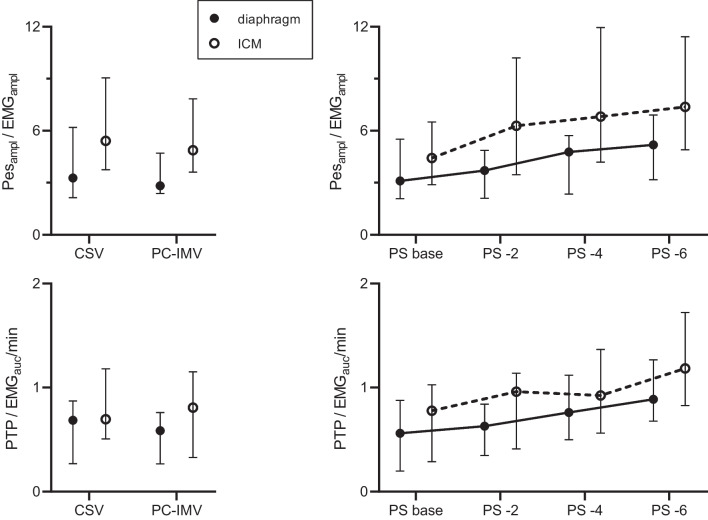
Neuro-mechanical coupling (NMC) of diaphragm and parasternal intercostals (ICM) during two different weaning methods (*left*) and reduction of pressure support (PS) level (*right*). Values are depicted as median (interquartile range). The number of subjects in which EMG and Pes parameters could be determined differed per muscle and ventilation mode or PS level. For the diaphragm *N* = 20 (median) and ICM *N* = 18 (median). *CSV* continuous spontaneous ventilation, *PC-IMV* pressure-controlled intermittent mandatory ventilation, *Pes*_*amp*_*/EMG*_*amp*_ esophageal pressure amplitude divided by EMG activity amplitude and PTP*/EMG*_*AUC*_*/min* pressure time product of esophageal pressure multiplied with the respiratory rate divided by integral of EMG signal over time during the inspiration multiplied with the respiratory rate

**Fig. 4:**
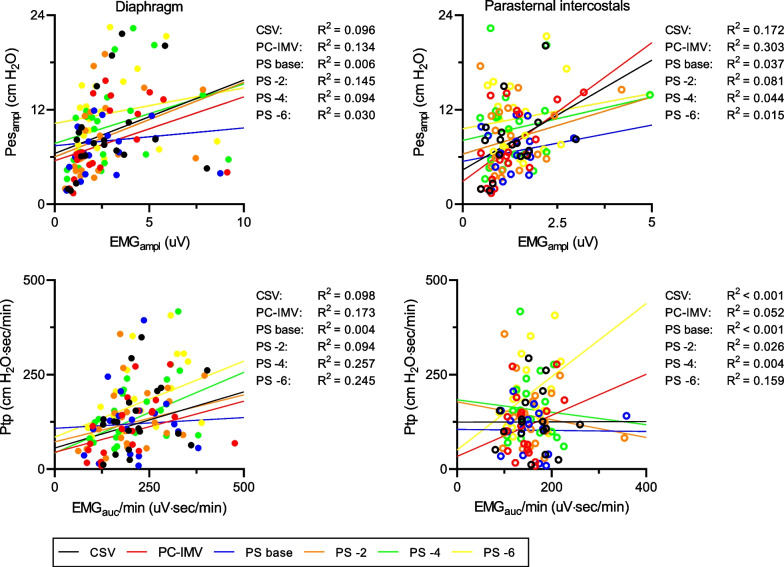
Correlation for the global population between inspiratory muscle activity of diaphragm (*left*) and parasternal intercostals (*right*), and inspiratory work of breathing in each condition of ventilator assistance. On the right side of each graph, the respective determination coefficient *R*^2^ is shown for each condition of ventilator assistance. *CSV* continuous spontaneous ventilation, *EMG*_*amp*_ EMG activity amplitude, *EMG*_*AUC*_*/min* integral of EMG signal over time during the inspiration multiplied with the respiratory rate, *PC-IMV* pressure-controlled intermittent mandatory ventilation, *Pes*_*amp*_ esophageal pressure amplitude, *PS* pressure support, and *PTP* pressure time product of esophageal pressure multiplied with the respiratory rate

Neuro-mechanical coupling of the diaphragm was estimated by a median Pes_ampl_/EMG_ampl_ of 3.3 and 2.8 cmH_2_O/µV, and median PTP/EMG_AUC_ × min^−1^ 0.69 and 0.59 cmH_2_O∙s∙min^−1^/µV during PS ventilation and PC-IMV, respectively. Neuro-mechanical coupling of ICM was described by a median Pes_ampl_/EMG_ampl_ of 5.4 and 4.9 cmH_2_O/µV, and median PTP /EMG_AUC_ × min^−1^ 0.70 and 0.81 cmH_2_O∙s∙min^−1^/µV during PS ventilation and PC-IMV, respectively. Neuro-mechanical coupling was comparable between PS ventilation and PC-IMV (Fig. 3). Estimates for neuro-mechanical coupling increased for diaphragm and ICM when PS was reduced, indicating that inspiratory muscles generated more pressure for 1 µV of EMG, as shown in Fig. 3. Diaphragm neuro-mechanical coupling estimates were median Pes_ampl_/EMG_ampl_ 3.1, 3.7, 4.8, and 5.2 cmH_2_O/µV for PS base, -2, -4, and -6 cm H_2_O, respectively; *p* = 0.05; and median PTP /EMG_AUC_ × min^−1^ 0.56, 0.63, 0.76, and 0.89 cmH_2_O∙s∙min^−1^/µV for PS base, -2, -4, and -6 cmH_2_O, respectively; *p* = 0.004. ICM neuro-mechanical coupling estimates were median Pes_ampl_/EMG_ampl_ 4.4, 6.3, 6.8, and 7.4 cmH_2_O/µV for PS base, -2, -4, and -6 cm H_2_O, respectively; *p* < 0.001 and median PTP /EMG_AUC_ × min^−1^ 0.78, 0.96, 0.92, and 1.18 cmH_2_O∙s∙min^−1^/µV for PS base, -2, -4, and -6 cm H_2_O, respectively; *p* < 0.001. Diaphragmatic EMG_amp_ and EMG_auc_/min were poorly correlated with Pes_amp_ (R^2^ < 0.15) and PTP (R^2^ < 0.26,) (Fig. 4). For the parasternal intercostals, comparable weak relationships were found between EMG_amp_ and Pes_amp_ (R^2^ < 0.31), and EMG_auc_/min and PTP (R^2^ < 0.06) (Fig. 4).

## Discussion

The main finding of this study is that it was possible to quantify the electrical activity of the diaphragm and parasternal intercostal muscles in mechanically ventilated children through transcutaneous recordings (sEMG). Breath-by-breath analysis showed a time-dependent relation between inspiratory sEMG and the ventilator pressurization reflected by a positive trigger time and almost neutral cycle time in PS ventilation. Furthermore, the electrical activity of both diaphragm and ICM increased in response to decreasing inspiratory level of assist on a cohort level.

### Neuro-respiratory drive

In clinical practice, monitoring of the electrical activity of inspiratory muscles may facilitate in identifying patient-ventilator asynchrony [20–22], diaphragm paresis [23], and central hypoventilation and apneas [24], titrating ventilatory support [25, 26], and assessing the use of (accessory) inspiratory and expiratory muscles in spontaneously breathing children [26–30]. Although absolute [30, 31] as well as normalized sEMG [25–28] values have been published in previous investigations, normal sEMG values of inspiratory muscles are still lacking. Therefore, it is difficult to interpret the sEMG values observed in this study. Theoretically, sEMG values can be interpreted in relation to the maximal sEMG obtained during a voluntary maximal effort, equal to EAdi [32]. However, voluntary maximal effort cannot reliably be obtained in critically ill children. In this study, we therefore normalized the inspiratory muscle activity to activity at baseline measurement for the interpretation of the neuro-respiratory drive.

To our best knowledge, this is the first study examining several neuro-respiratory drive parameters of the diaphragm and ICM in MV pediatric patients. We found that EMG_amp_ of both diaphragm and ICM, and diaphragmatic EMG_peak_ and EMG_AUC_/min increased in response to decreasing pressure support. In contrast, MacBean and colleagues only assessed EMG_peak_ of ICM and reported also higher values during less ventilatory support [29]. Others reported that (pre-term) infants and children failing extubation had a higher diaphragmatic EMG_peak_ and tonic EMG, both pre and post extubation [30]. In ventilated adults, it was shown that EMG_peak_ and EMG_AUC_ of both diaphragm and extradiaphragmatic inspiratory muscles increased in response to lower inspiratory support levels [25, 26].

Based on our results, EMG_amp_ is the most accurate neuro-respiratory drive parameter. For both inspiratory muscles, EMG_amp_ responded to increasing respiratory load on a group level. When reducing the level of inspiratory support, first diaphragmatic EMG_amp_ increased linearly whereas EMG_amp_ of ICM remained constant. Subsequently, diaphragmatic EMG_amp_ reached a plateau and EMG_amp_ of ICM increased exponentially at the lowest level of support. Therefore, our data suggests that when the maximum diaphragm capacity is reached, ICM will be increasingly recruited in case of a further PS reduction.

The neural respiratory drive is best represented by measured electrical activity of the diaphragm [33]. Recruitment of the accessory muscles is a well-known clinical sign of an increase in respiratory load. In both healthy subjects and ventilated patients, there is a hierarchy with respect to respiratory muscle recruitment [25]. In case of an increase in respiratory load, the diaphragm is immediately activated, followed by the chest wall muscles and subsequently by expiratory muscles [27, 28]. In our pediatric patients, we observed a similar recruitment pattern of the diaphragm and ICM represented by EMG_amp_.

Remarkably, in our study diaphragmatic EMG parameters showed values less than 100% of baseline. The higher levels of diaphragmatic electrical activity during baseline measurements might be explained by patients being agitated from instrumenting them for the data acquisition, since the diaphragm responds to an acute increase of respiratory load. However, accessory inspiratory muscles including ICM are recruited by a prolonged increased work of breathing, explaining possibly that EMG levels of ICM were not lower than baseline levels.

### Timing of respiratory muscles

In mechanically ventilated children, the patient ventilator interaction is often asynchronous [34–36]. We showed an increased cycle time during PC-IMV compared to CSV in both diaphragm and ICM, indicating a more asynchronous patient-ventilator interaction during PC-IMV. In PS ventilation, the timing of expiration is indirectly determined by the patient, i.e. the expiration trigger setting, instead of a set inspiration time as in PC-IMV, underlying a more synchronous interaction. In contrast to our finding, other research did not report a difference in cycle time between PC-IMV and CSV but we cannot easily explain this [36].

### Neuro-mechanical coupling

We found that NME indices for both diaphragm and ICM were affected by the level of assistance. Such a relation has also been described by the level by Essouri et al. [13] in pediatric patients when comparing the NME during mechanical ventilation and post extubation, using EAdi instead of surface EMG. Of note, Mortamet et al*.* [37] did not find significant changes in Pes_ampl_/EAdi_ampl_ before, during and after a spontaneous breathing trial. However, they also reported no increase in neuro-respiratory drive and respiratory effort, indicating that patients did not develop respiratory distress or fatigue during their spontaneous breathing trial. The dependency of NME indices on the level of muscle loading might be caused by the recruitment of accessory muscles [26], the increased efficiency of the diaphragm and ICM together or a combination of both. In addition to the diaphragm and ICM, other accessory inspiratory and expiratory muscles should be recorded to model the interaction between neuro-respiratory drive and respiratory effort accurately, in future research.

In each condition of ventilator assistance, a poor correlation was observed between neuro-respiratory drive and respiratory effort in the global population, suggesting muscle electrical activity is not synonymous with muscle contraction and force generation. In contrast, a strong linear correlation was previously described between EAdi and respiratory effort in different ventilator conditions in pediatric patients [13]. However, in that particular study measurement noise was reduced trough aggregation of similar breaths in their analysis. The NME index is highly variable between different patients but it is quite stable within a respiratory stable patient, regardless of whether the electrical activity of the diaphragm is recorded transcutaneous or transesophageal [14, 31, 38]. However, in case of respiratory distress or muscle fatigue, this relationship can change as indicated by our data.

### Limitations

Several limitations of our study need to be addressed. First, this study was designed a single center study potentially limiting the generalizability of our findings, although our unit is comparable to most large PICUs globally. Second, patients could be enrolled in our study when the attending physician deemed the patient eligible for weaning. Nevertheless, weaning is often not considered early enough in the course of MV in pediatric patients [39]. This may have led to a selection bias with the respiratory less stable patients not being included. Also, not all patients tolerated the lowest PS level in our study because of respiratory distress. Third, the study was not blinded but all signals were analyzed offline. From each enrolled subject, a single time period to be analyzed was manually selected. Subsequently those time periods were analyzed automatically using a custom-written Matlab script, making it unlikely that the results are affected by the unblinded nature of the study. Fourth, we found that in several patients no muscle activity could be detected from parasternal intercostals during particular ventilatory conditions or the whole study period. This could be caused by inactivity of the muscles possibly due to overassistance or oversedation, or low signal-to-noise ratio. Finally, we were not able to perform synchronized breath-by-breath analysis of EMG- and Pes-derived data as these physiological data was simultaneously recorded on two different non-synchronized devices.

## Conclusions

In summary, monitoring sEMG of parasternal intercostal muscles and diaphragm in the weaning phase of ventilated children is feasible and it might be helpful in a better understanding of the pediatric ventilation liberation process. We demonstrated that both neuro-respiratory drive and neuro-mechanical efficiency increase in response to lower inspiratory assistance.

## Supplementary Information

**Additional file 1: Table S1.** Absolute values electrical muscle activity of diaphragm and parasternal intercostals in two ventilation modes and at reducing PS levels.

## Data Availability

Data sharing requests will be considered by the research group upon written request to the corresponding author.

## Abbreviations

CSV: Continuous spontaneous ventilation
EAdi: Electrical activity of the diaphragm
EMG_amp_: Amplitude electrical muscle activity
EMG_mean_: Mean electrical muscle activity
EMG_peak_: Maximal electrical muscle activity
EMG_AUC_/min: Integral of EMG signal over time during inspiration multiplied per minute
FiO_2_: Fraction of inspired oxygen
ICM: Parasternal intercostal muscles
IQR: Interquartile range
MV: Mechanical ventilation
NME: Neuro-mechanical efficiency
PC/AC: Pressure controlled assist/control
PC-IMV: Pressure controlled-intermittent mandatory ventilation
PEEP: Positive end-expiratory pressure
Pes: Esophageal pressure
Pes_amp_: Tidal esophageal peak-to-trough during inspiration
PICU: Pediatric intensive care unit
PS: Pressure support
P-SILI: Patient self-inflected lung injury
PRISM: Pediatric RISK of Mortality
PTP: Pressure time product
sEMG: Surface electromyography
