# Supplementary material for: Surface electromyography to quantify neuro-respiratory drive and neuro-mechanical coupling in mechanically ventilated children
Source: Respir Res. 2023 Mar 13;24:77. doi: 10.1186/s12931-023-02374-w (PMC10010013; doi:10.1186/s12931-023-02374-w)
Supplement: Supplementary file 1 — Additional file 1: Table S1. Absolute values electrical muscle activity of diaphragm and parasternal intercostals in two ventilation modes and at reducing PS levels. [file 12931_2023_2374_MOESM1_ESM.docx]

**Surface electromyography to quantify neuro-respiratory drive and neuro-mechanical coupling in mechanically ventilated children**

Alette A. Koopman, MSc (1)

Jefta van Dijk, MD (1)

Eline Oppersma, PhD (2)

Robert G.T. Blokpoel, MD (1)

Martin C.J. Kneyber, MD, PhD FCCM (1, 4)

Additional file

**Table E1: Absolute values electrical muscle activity of diaphragm and parasternal intercostals in two ventilation modes and at reducing PS levels.**

|  |  | Ventilation modes | | | | Reducing PS level | | | | | | | |
| --- | --- | --- | --- | --- | --- | --- | --- | --- | --- | --- | --- | --- | --- |
|  |  | CSV | | PC-IMV | | PS base | | PS -2 | | PS -4 | | PS -6 | |
| Diaphragm | EMG_peak_ (µV) | 6.03 | [5.20-8.96] | 6.01 | [4.29-7.90] | 5.86 | [4.93-8.61] | 5.47 | [5.00-7.95] | 6.01 | [4.91-9.16] | 6.36 | [5.06-9.58] |
|  | EMG_ampl_ (µV) | 2.23 | [1.31-3.49] | 1.92 | [1.17-2.95] | 1.81 | [1.31-3.17] | 1.90 | [1.47-2.66] | 2.09 | [1.47-3.38] | 2.06 | [1.61-3.95] |
|  | EMG_mean_ (µV) | 4.95 | [4.20-6.85] | 4.91 | [3.40-6.36] | 4.87 | [4.09-6.70] | 4.79 | [3.93-5.87] | 5.24 | [4.13-6.73] | 5.35 | [4.02-6.86] |
|  | EMG_AUC_/min (µV×s/min) | 203 | [162-251] | 204 | [127-258] | 204 | [152-250] | 182 | [158-244] | 196 | [162-284] | 220 | [159-308] |
| ICM | EMG_peak_ (µV) | 5.18 | [4.54-5.78] | 4.83 | [4.33-5.25] | 5.18 | [4.22-5.56] | 4.96 | [4.30-5.52] | 5.05 | [4.42-5.69] | 4.96 | [4.46-5.62] |
|  | EMG_ampl_ (µV) | 1.27 | [0.97-1.57] | 1.19 | [0.81-1.54] | 1.27 | [0.90-1.73] | 1.04 | [0.84-1.51] | 1.29 | [0.87-1.87] | 1.50 | [1.07-1.98] |
|  | EMG_mean_ (µV) | 4.55 | [3.69-4.82] | 4.16 | [3.54-4.65] | 4.21 | [3.43-4.84] | 4.27 | [3.56-4.81] | 4.31 | [3.57-4.86] | 4.14 | [3.60-4.61] |
|  | EMG_AUC_/min (µV×s/min) | 159 | [139-184] | 140 | [124-164] | 155 | [121-174] | 156 | [137-176] | 156 | [135-176] | 151 | [126-184] |

*Abbreviations: CSV* continuous spontaneous ventilation,  *EMG_peak_* peak EMG activity, *EMG_amp_* EMG activity amplitude, *EMG_mean_* mean EMG activity level during one breath, *EMG_AUC_/min* integral of EMG signal over time during the inspiration multiplied with the respiratory rate, *ICM* intercostal muscles, *PC-IMV* pressure controlled intermittent mandatory ventilation, *PS* pressure support
Data are shown as median [interquartile range].
